# *CBFB::MYH11* Fusion Located on a Supernumerary Ring Chromosome 16 in Pediatric Acute Myeloid Leukemia: Diagnostic Challenges and Prognostic Implications

**DOI:** 10.3390/genes16111283

**Published:** 2025-10-29

**Authors:** Changqing Xia, Melissa Acquazzino, Pamela A. Althof, Marilu Nelson, Rachel A. Harris, Joanna R. Spaulding, Joseph D. Khoury, Zhenya Tang

**Affiliations:** 1 Warren G Sanger Human Genetics Laboratory, Department of Pathology, Microbiology and Immunology, University of Nebraska Medical Center and Nebraska Medicine, Omaha, NE 68918, USA; paalthof@nebraskamed.com (P.A.A.); marnelson@nebraskamed.com (M.N.); raharris@nebraskamed.com (R.A.H.);; 2 Hematology/Oncology, Department of Pediatrics, University of Nebraska Medical Center, Omaha, NE 68918, USA; macquazzino@childrensnebraska.org; 3 Hematopathology, Department of Pathology, Microbiology and Immunology, University of Nebraska Medical Center and Nebraska Medicine, Omaha, NE 68918, USA; jkhoury@unmc.edu

**Keywords:** *CBFB::MYH11*, ring chromosome, fluorescent in situ hybridization, Karyotyping, Chromosome microarray, next generation sequencing

## Abstract

We report a unique pediatric acute myeloid leukemia (AML) case characterized by a *CBFB::MYH11* fusion located on a supernumerary ring chromosome 16. Following diagnosis through comprehensive blood and bone marrow assays, the patient was enrolled in the Children’s Oncology Group (COG) study AAML1831 and randomized to the experimental treatment arm (Arm B). She received induction chemotherapy with CPX-351 (liposomal daunorubicin and cytarabine), gemtuzumab and ozogamicin (GO), and the cardioprotectant dexrazoxane and achieved complete remission (CR). The patient completed the treatment with sustained CR for 18 months. This case represents a rare cytogenetic phenomenon that is not well-documented in the current literature. Through a review of relevant publications, we contextualize this case within the spectrum of core binding factor AML (CBF-AML), highlighting diagnostic approaches, treatment strategies, and prognostic implications, particularly in cases involving atypical *CBFB::MYH11* fusions. The durable remission observed in this patient, despite the unusual cytogenetic presentation, provides valuable insights into therapeutic management. This report underscores the cytogenetic and molecular heterogeneity of *CBFB::MYH11* AML and emphasizes the importance of comprehensive genetic profiling using advanced techniques such as chromosomal microarray and next-generation sequencing.

## 1. Introduction

Acute myeloid leukemia with *CBFB::MYH11* fusion (*CBFB::MYH11* AML) is a subgroup of core binding factor AML (CBF-AML), typically resulting from either inv(16) (p13.1q22) or t(16; 16) (p13.1; q22), and accounts for approximately 12% of all pediatric AML cases [1]. This fusion transcript disrupts normal hematopoietic differentiation by forming a chimeric protein that interferes with the core binding factor (CBF) complex, a critical regulator of normal hematopoiesis [2]. The CBF complex in AML with inv(16) or t(16;16) consists of a heterodimeric transcription factor composed of DNA-binding CBFA subunits and a non-DNA-binding CBFB subunit that stabilizes the complex [3]. The disruption of this complex leads to maturation arrest and leukemogenesis through aberrant transcriptional programming [4,5,6].

The prognosis of CBF-AML has traditionally been classified as favorable, particularly when treated with cytarabine-based intensive chemotherapy regimens, presented as high complete remission (CR) rates and relatively improved survival outcomes compared to other AML subtypes [7,8]. However, despite this overall favorable categorization, approximately 30~40% of CBF-AML patients still experience relapse with median follow-up time of 3.5 years [9], which highlights the molecular heterogeneity within this disease category and underscores the importance of refined prognostic stratification.

The landscape of *CBFB::MYH11* transcript variants reveals significant molecular diversity with prognostic implications. To date, over 12 *CBFB::MYH11* transcripts have been identified, with type A (*CBFB::MYH11*/*E5-E33*, indicating a fusion between *CBFB exon 5* and *MYH11 exon 33*) being the most prevalent representing 79~87% of cases, followed by types E (*CBFB::MYH11/E5-E28*, 5–9%) and D (*CBFB::MYH11/E5-E29*, 3–10%) [10,11,12]. Certain rare fusion transcripts, such as type I (*CBFB::MYH11/E5-E8*), have been reported mostly as case reports, and they not only present diagnostic challenges due to their rarity and a wide spectrum of fusion patterns but also may have distinct biological behaviors [12,13]. A recent study by Huang et al. has demonstrated that the types of *CBFB::MYH11* transcripts are associated with clinical outcomes, with type A associated with inferior event-free survival compared to other variants [14].

The presence of additional genetic abnormalities can further complicate the prognostic landscape of CBF-AML. For example, *KIT* mutations occur in approximately 30% of CBF-AML cases and have been associated with increased relapse risk [14]. Similarly, *FLT3* mutations, particularly *FLT3-TKD*, are detected in approximately 3.4% of pediatric CBF-AML cases and may influence therapeutic responsiveness [15]. The co-occurrence of *BCR::ABL1* with *CBFB::MYH11* represents an exceptionally rare phenomenon, which mostly belongs to blast phase of chronic myeloid leukemia and is typically associated with poor outcomes [16,17]. Rare cases with *CBFB* rearrangement but partnering with other genes than *MYH11* have also been reported [18,19,20].

Ring chromosomes represent structurally abnormal chromosomes whose origins usually cannot be determined through conventional cytogenetic testing and require additional more specific and advanced assays. In our case, the occurrence of *CBFB::MYH11* fusion on a supernumerary ring chromosome 16 represents a novel cytogenetic alteration. Several techniques, such as metaphase FISH, SNP microarray and next-generation sequencing (NGS) were utilized to characterize the chimeric gene location and the possible genomic buildup. To our knowledge, this is the first case with *CBFB::MYH11* fusion located on a supernumerary ring chromosome 16. This case report aims to address this knowledge gap by presenting a pediatric case with this unique genetic rearrangement who responded well to the treatment and achieved sustained remission, providing valuable insights into disease biology, diagnosis and clinical management.

## 2. Case Presentation

A previously healthy 8-year-old female presented with the symptoms of abdominal pain and epistaxis and was found to have anemia and thrombocytopenia on lab work. Her initial complete blood count (CBC) exhibited a white blood cell count of 13.14 × 10^9^/L, hemoglobin of 5.1 g/dL, and platelets of 38 × 10^9^/L. Her manual differential showed 50% of blasts. Flow cytometry on peripheral blood revealed a diagnosis of acute myeloid leukemia (AML) with the blast cells expressing CD11b, CD11c, CD15, CD13, CD24, CD33, CD34, CD117, CD123 and HLA-DR. Cytogenetic analysis for peripheral blood was not performed because of the limited amount of sample. However, a subsequent bone marrow biopsy was conducted, and the bone marrow specimen was submitted for genetic and molecular testing (For a newly diagnosed AML, peripheral blood and bone marrow samples are generally considered equivalent in terms of diagnostic significance, particularly when circulating blasts are present). Diagnostic lumbar puncture was negative for central nervous system disease.

**Figure 1:**
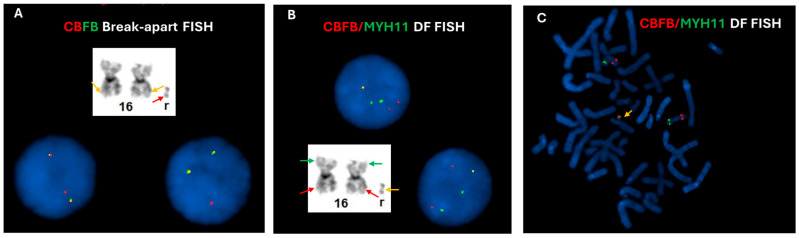
FISH assessment for *CBFB* rearrangement on bone marrow cultures. (**A**) Interphase FISH using *CBFB* break-apart probes revealed two normal, intact fusion signals (yellow signals) which likely located on the morphologically normal chromosomes (orange arrows), and one isolated *5′CBFB* (red signal) which is likely located on the ring chromosome (red arrow), possibly indicating an unbalanced *CBFB* rearrangement. (**B**) Interphase FISH using *CBFB* (red)::*MYH11* (green) dual fusion (DF) probes showed two red and two green signals, along with one fusion signal (yellow), consistent with a variant *CBFB::MYH11* rearrangement. (**C**) Metaphase FISH analysis using *CBFB* (red)::*MYH11* (green) dual fusion (DF) probes demonstrated that the fusion signal (yellow arrow) is located on the ring chromosome, while the separate red and green signals were located on 16p and 16q in two normal chromosomes 16, respectively.

Chromosomal analysis of the bone marrow using GTG banding technique revealed a hyperdiploid female karyotype with a supernumerary ring chromosome, described as 47, XX, +r in all 20 cells analyzed. Interphase FISH using *CBFB* break-apart (BAP) probe (Abbott Molecular Inc. Abbott Park, IL, USA) demonstrated an atypical signal pattern, characterized by two intact *CBFB* signals (yellow) and one isolated 5′ *CBFB* (red) signal in 164 out of 200 cells analyzed (Figure 1A). To further characterize the atypical signal pattern, the *CBFB::MYH11* dual color dual fusion (DF) probe set (Metasystems, Medford, MA) was subsequently employed with an abnormal finding of two red, two green and one fusion (2R2G1F) in 168 out of 200 cells analyzed (Figure 1B), which is considered an atypical positive result for *CBFB::MYH11* rearrangement. Interestingly, the *CBFB::MYH11* fusion signal was revealed to be located on the ring chromosome through metaphase FISH analysis by selectively analyzing five metaphases with *CBFB::MYH11* fusion signal (Figure 1C).

**Figure 2:**
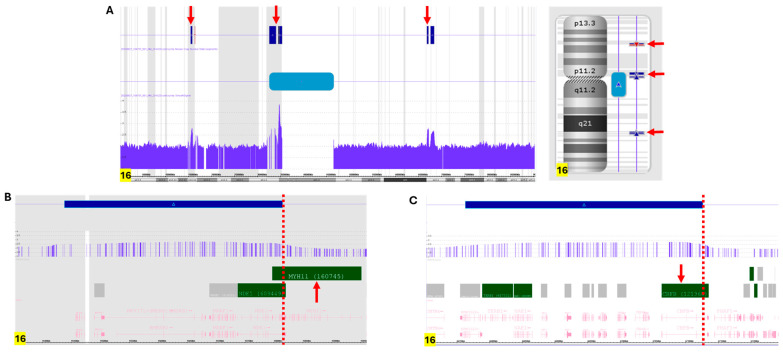
SNP microarray analysis of fresh bone marrow cells. (**A**) SNP microarray analysis revealed several duplicated segments on chromosome 16 (pointed by red arrows): one at 16p13.11 (arr[GRCh37] 16p13.11(15,438,165_15,814,747)x3) encompassing the *MYH11* gene, one at 16q22.1 (arr[GRCh37] 16q22.1(66,762,847_67,125,895)x3) encompassing the *CBFB* gene, and a larger region spanning the centromere (arr[GRCh37] 16p11.2q11.2(32,511,179_46,534,977)x3), represented by a blue bar. (**B**) Detailed analysis of the duplicated 16p13.11 region indicated that the 3′*MYH11* (pointed by red arrow), near exon 33 (shown by red dotted line), is duplicated. (**C**) Detailed analysis of the duplicated 16q22.1 region indicated that the 5’*CBFB* (pointed by red arrow), near exon 5 (shown by red dotted line), is duplicated.

Concurrent SNP microarray (Affymetrix CytoScan HD from Themo-Fisher, Waltham, MA, USA) analysis revealed three duplicated segments of chromosome 16, including a 377 kb segment affecting 16p12.11 which partially encompasses 3′ *MYH11* (GRCh37:15438165-15814747), a 363 kb segment involving 16q22.1 which partially encompasses 5′ *CBFB* (GRCh37: 66762847-67125895), and a large duplicated segment (14.25 Mb) likely spanning the centromere of chromosome 16 (GRCh37: 32511179-46534977) (Figure 2A). These findings suggest that the ring chromosome is composed of rearranged segments from chromosome arms 16p and 16q, resulting in the *CBFB::MYH11* fusion. Based on the information of the coordinates by SNP microarray, a type A (*CBFB::MYH11/E5–E33*) transcript can be postulated using the UCSC Genome Browser v. GRCh37. The presence of an intact centromere may explain the faithful chromosome segregation and transmission of this ring chromosome 16 during cell division (Figure 2A) [21].

Additionally, next-generation sequencing (NGS) was performed on the bone marrow specimen. Several pathogenic gene mutations, including *NRAS p.G13D*, *NOTCH4 splice site 73+1G > A*, KMT2D p.G2892Afs*18, *EGFR p.Y727C*, NF1 p.I679Dfs*21, and KDM6A p.R1111Gfs*40 were identified. A *CBFB::MYH11* fusion was also detected by RNA sequencing through the NGS platform, Foundation One Heme comprehensive genomic profiling panel (Cambridge, MA, USA).

The patient was enrolled on the Children’s Oncology Group (COG) study AAML1831 to begin treatment. She was randomized to the experimental arm (Arm B) of the study and received CPX-351 (study drug, liposomal daunorubicin and cytarabine), gemtuzumab ozogamicin, and the cardioprotectant dexrazoxane for her induction chemotherapy. Repeated bone marrow on day 36 of induction therapy was negative for disease by both local flow cytometry and minimal residual disease flow cytometry assessment at Hematologics, Inc. (Seattle, WA, USA), the AML reference laboratory. The patient completed treatment on Arm B of COG AAML1831. In total, she received 5 cycles of chemotherapy. Treatment was complicated by a rash from the study drug, malnutrition, cellulitis, typhlitis, clostridium difficile colitis, methicillin-resistant *Staphylococcus aureus* bacteremia, and acute kidney injury. Her end-of-treatment bone marrow evaluation showed continued remission. She continues to be followed in the Oncology Clinic and is now 18 months after completion of treatment. She is clinically doing well, remains in remission, and is without any late side effects of her cancer treatment.

## 3. Discussion

The classical model for ring chromosome formation involves terminal double-strand DNA breaks followed by end-to-end fusion, often accompanied by loss of genetic materials distal to the breakpoints [22]. Alternative mechanisms include telomere–telomere fusion without significant DNA loss or breakage–fusion–bridge (BFB) cycles potentially leading to chromosomal instability [23]. These mechanisms may operate sequentially, leading to dynamic ring chromosome structures prone to progressive enlargement, gene amplification, loss or complex rearrangements [24,25]. Ring chromosomes represent a clinically significant cytogenetic abnormality in AML, and the instability of ring chromosomes has been implicated in clonal evolution and disease progression [26,27]. Thus, ring chromosomes may be associated with distinct diagnostic and prognostic implications in AML.

Ring chromosomes in AML are rare, with an estimated frequency of <1% among all cytogenetically abnormal AML cases [28]. They have been reported involving a wide range of chromosomes, including chromosomes 7, 11, 13, 17, and 21, with ring chromosome 7 and 11 being relatively more frequent [29]. In many cases, ring chromosomes occur as part of a complex karyotype conferring an adverse prognosis [26,27,30]. Ring chromosomes are also thought to drive genomic instability through ongoing BFB cycles and intrachromosomal recombination, leading to further clonal heterogeneity [22,31]. This instability may facilitate the emergence of resistant subclones under therapeutic pressure, contributing to treatment failure and relapse [30]. Indeed, studies in myeloid malignancies broadly suggest that the presence of ring chromosomes correlates with poor response to induction chemotherapy and shorter overall survival [32]. Nonetheless, a subset of case reports has described AML patients with ring chromosomes achieving remission [33,34], suggesting that prognosis may be context-dependent, influenced by the specific chromosome involved, the size of the ring, and accompanying genetic lesions, as well as the therapeutic regimen employed. More systematic studies are required to clarify whether certain ring chromosomes carry distinct biological and clinical consequences. Isolated ring chromosomes without additional major cytogenetic abnormalities are extremely rare, such as the case presented here, making it more challenging to unravel their independent prognostic effect than those with other co-existing aberrations.

Cytogenetic identification of ring chromosomes poses significant diagnostic challenges. Conventional G-banding may reveal atypical circular structures, but the small size (such as our current case), variable morphology, and tendency of rings to undergo mitotic instability often complicate karyotypic analysis [35]. Moreover, ring chromosomes may be cryptic or misclassified as marker chromosomes, requiring complementary molecular cytogenetic and cytogenomic tools such as fluorescence in situ hybridization (FISH), chromosome microarray (CMA), or next-generation sequencing (NGS) as well as optic genome mapping (OGM) for precise characterization [30,36,37]. Indeed, the small ring chromosome 16 presented in our case was only able to be characterized by combined techniques including karyotyping, interphase and metaphase FISH, SNP microarray and NGS. Although Optical Genome Mapping (OGM) was not utilized in the diagnostic workup of this case, it could potentially aid in identifying the *CBFB::MYH11* rearrangement. However, it is important to note that while OGM may detect the presence of *CBFB::MYH11* rearrangement, it does not provide precise information regarding its chromosomal location. This limitation underscores the importance of integrating cytogenetic and cytogenomic techniques for comprehensive characterization. More intriguingly, these techniques enabled not only the identification of the critical AML-associated fusion gene, *CBFB::MYH11*, located on a small ring chromosome 16, but also the classification of molecular subset of this fusion gene (type A).

The presence of the *CBFB::MYH11* fusion within a supernumerary ring chromosome represents a novel cytogenetic context for this well-established AML subtype. In our newly diagnosed pediatric case, the localization of this fusion on a ring chromosome raises compelling questions regarding disease pathogenesis. Notably, despite this atypical genetic configuration, the patient has achieved and sustained CR, as confirmed by flow cytometric MRD testing. The outcome underscores the therapeutic and prognostic implications for CBF-AML cases with unconventional cytogenetic abnormalities.

The excellent treatment response observed in our patient may be attributed to several key factors: (1) the favorable risk similar to conventional *CBFB::MYH11* AML; (2) a rapid and dramatic reduction in leukemic burden following induction chemotherapy; (3) incorporation of gemtuzumab ozogamicin (GO) into the treatment regimen, which may have mitigated the potential adverse effects associated with the ring chromosome; and (4) the use of intensive consolidation therapy across multiple cycles, which likely contributed to the eradication of residual leukemic cells and counteracted the potential negative prognostic impact of the ring chromosome harboring the *CBFB::MYH11* fusion and the concurrent pathogenic gene mutations including *NRAS* mutation and other relatively uncommon gene mutations shown in the section above identified through next-generation sequencing. *RAS* activating point mutations are present in 10–30% of myeloid malignancies and associated with a proliferative phenotype, and poor clinical outcome in general [38].

While our patient did not exhibit the classic myelomonocytic differentiation with increased bone marrow eosinophiles typically associated with inv(16) AML, recent studies have highlighted the growing recognition of morphological and clinical heterogeneity within *CBFB::MYH11* AML [14]. Traditionally classified under the French-American-British (FAB) M4Eo subtype, this leukemia is known for its characteristic eosinophil-rich marrow. However, emerging evidence suggests that a subset of patients estimated at approximately 15% of *CBFB::MYH11* AML cases may present without significant eosinophilia, complicating morphological diagnosis [12]. This underscores the importance of specific diagnostic testing in identifying cryptic fusions or rare subsets of *CBFB::MYH11* AML that may otherwise be overlooked by morphological, conventional cytogenetic and molecular testing.

The clinical implications of *CBFB::MYH11* fusion localized to a ring chromosome remain uncertain due to the rarity of such cases. However, considerations emerge: (1) comprehensive genetic profiling including chromosomal microarray, optical genome mapping, and next-generation sequencing is essential to detect atypical presentations; (2) MRD monitoring using advanced technologies should guide therapeutic decisions regardless of cytogenetic complexity; and (3) prospective registration of rare cytogenetic variants in international databases would facilitate a better understanding of their prognostic significance.

From a biological standpoint, the localization of the *CBFB::MYH11* fusion to a supernumerary ring chromosome 16 may influence gene expression through alterations in chromatin architecture or the creation of altered gene expression profiling. Transcriptomic analyses have shown that different *CBFB::MYH11* fusion variants establish distinct transcriptional programs, with differential enrichment of hematopoietic stem cell self-renewal genes and early hematopoiesis pathways [14]. Whether the ring chromosome context further modifies this transcriptional landscape remains an open question and warrants further investigation.

In addition, constitutional ring chromosomes have been reported in identical twins who developed acute lymphoblastic leukemia at 11 and 15 years of age, respectively, following the acquisition of distinct molecular genetic alterations [39]. It remains unclear whether the small ring chromosome 16 observed in our case arose constitutionally and later contributed to leukemogenesis through secondary genetic events, such as the *CBFB::MYH11* fusion. Unfortunately, due to the unavailability of the patient, this question cannot be definitively resolved. Nonetheless, it represents a plausible hypothesis worthy of consideration.

## 4. Conclusions

This case report underscores the genetic heterogeneity of *CBFB::MYH11* AML and describes a rare cytogenetic presentation in pediatric AML, where the *CBFB::MYH11* fusion resides within a supernumerary ring chromosome 16. Despite its atypical configuration, the case demonstrates an excellent treatment response and sustained remission. Importantly, the presence of *CBFB::MYH11* fusion within a ring chromosome highlights the need for advanced cytogenetic, cytogenomic and molecular techniques to ensure accurate detection and classification. The prognostic impact of such atypical presentations may be influenced by co-occurring molecular alterations and the intensity of treatment. In the present case, the incorporation of gemtuzumab ozogamicin (GO) and multiple cycles of consolidation chemotherapy likely contributed to the favorable outcome. Regardless of cytogenetic complexity, MRD-guided therapy remains central to disease management. Finally, the registration of rare cytogenetic variants in international databases will be essential to better understand their prognostic significance and guide future therapeutic strategies.

## Data Availability

The original contributions presented in this study are included in the article. Further inquiries can be directed to the corresponding authors.
